# Police legitimacy and procedural justice for children and youth: a scoping review of definitions, determinants, and consequences

**DOI:** 10.3389/fsoc.2024.1409080

**Published:** 2024-09-25

**Authors:** Jessica C. M. Li, Serena Y. Zhang, Ivan Y. Sun, Albert S. K. Ho

**Affiliations:** ^1^Department of Applied Social Sciences, The Hong Kong Polytechnic University, Kowloon, Hong Kong SAR, China; ^2^Department of Sociology and Social Work, Aalborg University, Aalborg, Denmark; ^3^Department of Sociology and Criminal Justice, University of Delaware, Newark, DE, United States

**Keywords:** legitimacy, procedural justice, compliance, youth, police, trust

## Abstract

**Introduction:**

Understanding police legitimacy among children and youth is important for building a just and democratic society. Although the volume of studies on police legitimacy among underaged persons has grown in recent decades, the findings on the relationships between police legitimacy and procedural justice and their definitions, associated determinants, and consequences remain heterogeneous across studies and across political and legal contexts. Given these heterogeneities, the conclusions and implications generated by this research are far from comprehensive.

**Method:**

This scoping review offers readers a comprehensive and comparative understanding of this topic by answering the following questions. (1) How can we *define* police legitimacy and procedural justice for children and youth? (2) What are the *determinants* of police procedural justice and legitimacy for children and youth? (3) What are the *consequences* of police procedural (in)justice and (il)legitimacy for children and youth? (4) Among children and youth, who are the *vulnerable groups* receiving less legitimate and unjust treatment from the police? A scoping review of the literature published between January 1, 1990 and May 31, 2022 was conducted based on four databases: PubMed, Web of Science, Scopus, and ProQuest. Guided by the scoping review screening framework proposed by Arksey and O’Malley, that is, the Preferred Reporting Items for Systematic Reviews and Meta-Analysis guidelines, and the checklist provided by the Joanna Briggs Institute for quality assessment, 47 publications, consisting of 38 quantitative studies and 9 qualitative studies, were retained in the final sample.

**Results:**

The results synthesize the operational and subjective interpretations of police legitimacy offered by the respondents in the studies reviewed which is followed by the discussion of conceptual and measurement issues. The key correlates of police legitimacy identified in these studies were police procedural justice and behavior, followed by experience and contact with the police, relationships with other authority figures, and personal competence in moral reasoning and self-control. In addition to compliance and cooperation, cynicism, trust, and health were related to police (il)legitimacy.

**Discussion:**

We argue that in addition to building and maintaining police legitimacy, it is vital to remedy the negative consequences of injustice in police–youth encounters.

**Systematic Review Registration:**

https://inplasy.com/inplasy-2024-9-0064/, INPLASY202490064.

## 1 Introduction

Police procedural justice and legitimacy have been the focal concerns of many policing studies globally. Following Tyler’s (1990) seminal work Why People Obey the Law, there was an explosion in this vein of research (Tankebe, 2013). The core assumption of legitimacy research is that when people perceive that they are being treated with respect and fairness (i.e., perceived procedural justice) in their encounters with the police, they are likely to regard the police as legitimate and apt to comply and cooperate with them. Procedural justice is not the only mechanism to garner citizens’ compliance and cooperation. However, compared with instrumental compliance, procedural justice emphasizes individuals’ voluntary self-regulation that is more effective and durable (Hough, 2013; Jackson et al., 2021).

Police legitimacy is particularly relevant to children and youth, partly because they have more frequent contacts with the police as both offenders and victims (Bottoms, 2006). Unpleasant experiences with the police may have undesirable effects on youth trust in the criminal justice system. Unfortunately, young people’s perceptions of the police are becoming increasingly negative and have reached a decades-long low (Fine et al., 2022). Moreover, youth are often positioned as a group of “permanent suspects” (McAra and McVie, 2005, p. 9), highlighting the potential tensions between youth and the police. Understanding adolescents’ perceptions of police legitimacy may help facilitate better police–youth encounters. However, most investigations of police procedural justice have used samples of adult and college-age populations. More attention should be given to youth populations (Trinkner and Cohn, 2014). Virtually, adults reported more positive attitudes toward police procedural justice than youth (Murphy, 2015). Research also demonstrated that perceptions of police legitimacy followed a U-shaped curve, declining during adolescence, reaching its lowest point around age 18, and improving during the transition to young adulthood (Fine et al., 2021). Thus, whether young people have the same understanding of legitimacy and the same responses to procedural justice as adults is uncertain and deserves further investigation (Gau, 2011; Saarikkomäki, 2016).

This scoping review of police legitimacy among children and youth makes three contributions. First, as the definitions and measures of police legitimacy are heterogeneous and subject to debate (Varet et al., 2021), this scoping review can help readers grasp some ideas of the issues involved in researching police legitimacy across different social, legal, and cultural contexts. Second, the police can only garner compliance and cooperation from people who trust legal authorities. This scoping review can serve as a basis for developing policies and practices to enhance youth’s trust and confidence in the police. Knowing the determinants and consequences of adolescents’ views of police procedural justice and legitimacy is a vital reference for police management, training, and social interventions for youth and their families. Finally, this study is one of the first reviews of the quantitative and qualitative methods used to examine youth’s perceptions of police legitimacy.

## 2 Method

Unlike systematic reviews, which focus on a precise question about the effectiveness/evaluation of an intervention or relationships between well-defined concepts, a scoping review maps the literature to identify the key concepts, gaps, and sources of evidence that inform practice, policy making, and research (Daudt et al., 2013). The data reviewed in this study were heterogeneous because both qualitative and quantitative studies were included, thereby excluding the use of meta-analysis. As this review aimed to clarify the definitions, determinants, and consequences of police procedural justice/legitimacy, a scoping review was employed to achieve the research objective. The approach was underpinned by Arksey and O’Malley’s (2005) five-stage framework: (1) identifying the initial research questions, (2) locating relevant studies, (3) selecting studies, (4) charting the data, and (5) collating, summarizing, and reporting the results. These steps were adopted in this review of policing legitimacy among children and youth.

### 2.1 Identifying research questions about police legitimacy and procedural justice for children and youth

This review explored the key components of adolescents’ and youngsters’ perceptions of police legitimacy, thus contributing to a comprehensive, precise, and concise understanding of the relationships and dynamics between the police and the underaged. Specifically, we posed the following initial questions to guide the search. (1) How can we *define* police legitimacy and procedural justice for children and youth? (2) What are the *determinants* of police procedural justice and legitimacy for children and youth? (3) What are the *consequences* of police procedural (in)justice and (il)legitimacy for children and youth? (4) Among children and youth, who are the *vulnerable groups* receiving less legitimate and unjust treatment from the police?

### 2.2 Locating relevant studies

**Figure 1:**
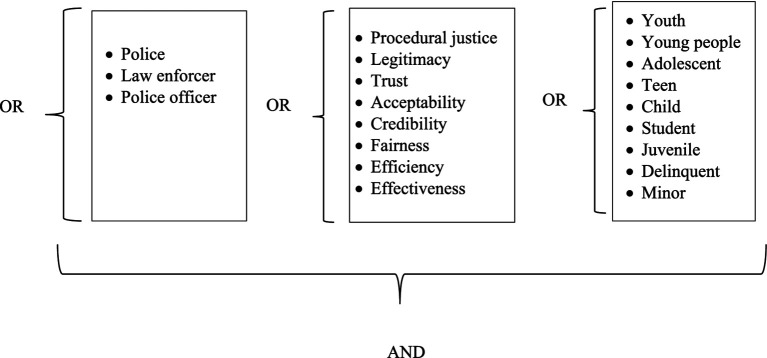
Keywords and Boolean operators used for article searching.

We searched four databases for relevant studies: PubMed, Web of Science, Scopus, and ProQuest. These databases were selected based on the following criteria: (1) they covered social/behavioral science and health studies literatures, (2) they had “advanced search” options, allowing keyword searches with categories (see Figure 1), and (3) some of them were used in previous scoping reviews of legitimacy in prisons (Ryan and Bergin, 2022) and legitimacy in traffic rule compliance (Varet et al., 2021). The timeframe for the search was from January 1, 1990, to May 31, 2022. As the main theoretical framework for our research was the model developed by Tyler and colleagues in the 1990s (Tyler, 1990; Tyler and Lind, 1992), we set the start date for our literature search after the theoretical model was proposed.

Based on the research questions, three groups of keywords were used for searching (see Figure 1). In Figure 1 (from left to right), the three categories of keywords refer to the executor of the central concept (i.e., police), the central concept (i.e., procedural justice and legitimacy), and the target population (i.e., youth). The Boolean operators AND, OR, NOT, OR and NOT were also specified between the three groups of keywords in the search. The keywords within each category were interchangeable; thus, the Boolean operator “OR” was used. Meanwhile, the Boolean operator “AND” was employed to ensure that at least one keyword from each group was present in each result. The publications that included these combinations of keywords in their titles, abstracts, or keywords were the focus of this study.

Publications were included if they met each of the following criteria: (1) were scholarly refereed journal articles (book chapters, dissertation/thesis, etc. were excluded), (2) were empirical studies using quantitative, qualitative, or mixed method approaches (reviews, proceedings, letters to the editor, newsletters, etc. were excluded); and (3) were published in English. Overall, 1,863 references were found. After removing duplicate entries, 984 references were retained in the initial sample.

### 2.3 Selecting studies (i.e., inclusion and exclusion criteria, quality appraisal)

**Figure 2:**
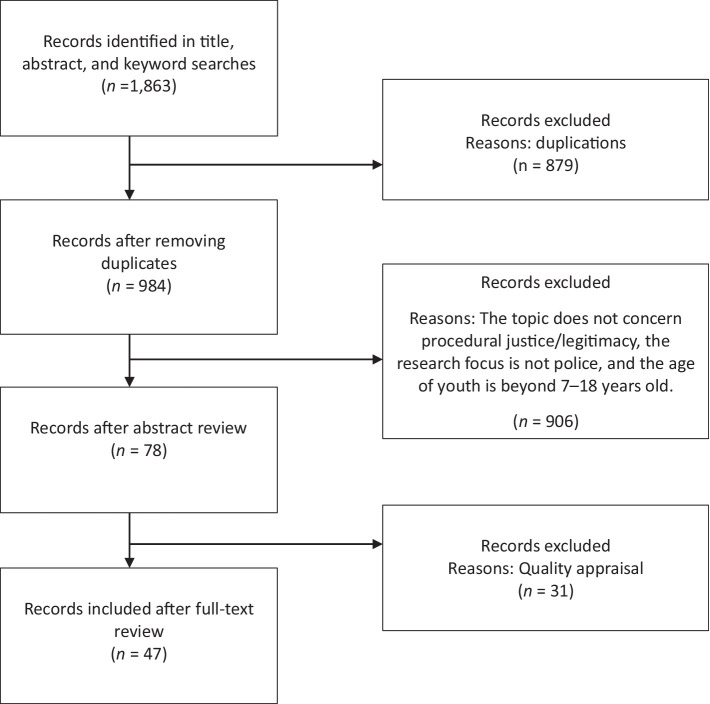
Preferred reporting items for systematic reviews and meta-analysis (PRISMA).

The third stage involved selecting the studies to be included in this review. Using the Preferred Reporting Items for Systematic Reviews and Meta-Analysis (PRISMA) guidelines (Liberati et al., 2009), the article selection was conducted manually in three rounds (see Figure 2). First, the second author independently classified the 984 articles into three categories: (1) articles beyond the scope of this review of police legitimacy (*N* = 799), for example, the focus was not on police officers exclusively nor procedural justice and legitimacy, (2) articles relevant to the scope (focus) of this review, but in which the age of the participants was outside the range of 7–18 years old (*N* = 107), and (3) articles considered relevant to the scope (focus) of this review in which the age of the participants was within the target range of 7–18 years old (*N* = 78). Only the 78 papers of the last category were included in the next step of the assessment.

In the second round, the first author conducted a quality assessment of the 78 articles identified in the first round, and the second author categorized the articles into two types (quantitative and qualitative studies) across five groups, including cross-sectional studies, qualitative studies, quasi-experimental studies, cohort studies, and randomized controlled trials. The Joanna Briggs Institute (JBI) is an evidence-based organization formed to develop methodologies and guidelines for conducting systematic or scoping reviews (Munn et al., 2014). It provides a checklist of 8–13 questions for evaluating the quality of studies with different research designs (see Joanna Briggs Institute, 2017, for full descriptions of the items in each checklist). For example, the items for assessing the cross-sectional studies include the following questions: (1) Were the criteria for inclusion in the sample clearly defined? (2) Were the study subjects and the settings described in detail? (3) Was the exposure measured validly and reliably? (4) Were objective, standard criteria used for measuring the condition? (5) Were confounding factors identified? (6) Were strategies to deal with confounding factors stated? (7) Were the outcomes measured validly and reliably? (8) Was appropriate statistical analysis used?

Based on the critical appraisal tools for assessing different research designs, the first two authors independently reviewed each article by answering either “yes, no, unclear, or not applicable (NA)” to each question in the checklist. Articles were removed if more than 20% of the questions in the checklist were answered “no, unclear, or NA.” Approximately 25 publications from the five groups of articles were dropped. The agreement rate in the first round was 84.62%. The two authors discussed the divergent decisions in the last round and reviewed these articles. After this step, 47 references remained (including 38 quantitative and 9 qualitative studies; see Tables 1, 2), and the agreement rate was 99%. The quantitative studies used cross-sectional, longitudinal, and experimental designs, and the qualitative studies used individual interviews, focus group interviews, and observation methods.

**Table 1:** Quantitative studies (including cross-sectional, longitudinal, and experimental methods) (*n* = 38).

	Country	*N* (% males)	Mean age	Sample	Methods	Relevant findings
Akinlabi (2017)	Nigeria	305 (55.7%)	15.13	School students	Cross-sectional survey design	Young people who perceived the police as using fair procedures were likely to see the police as legitimate. Young people with few experiences with the police will likely ascribe high legitimacy to the police.
Baz and Fernandez-Molina (2018)	Spain	2,041 (49.1%)	15.33	School students	Cross-sectional survey design	Police legitimacy perceptions are not only influenced by procedural justice but also by parental monitoring, school attachment, and delinquent peers. Youths who had some contact with the police due to the illegal acts committed tend to attribute little legitimacy to the police as an authority.
Cavanagh and Cauffman (2019)	USA	634 (50% sons)	15.35 (son) vs. 46.32 (mother)	Male juvenile offenders and their mothers	Longitudinal survey design	A mother’s attitude toward the police may outweigh a youth’s experience (via arrests) with the justice system in determining the trajectories of his attitudes toward the police.
Cohn et al. (2012)	USA	348 (34.5%)	14.28	School students	Longitudinal survey design	Legal (but not moral) reasoning was associated with both parental and police legitimacy—which were associated with RVB (rule-violating behavior) via the mediating influence of legal reason (normative status).
Dirikx et al. (2013)	Belgium	356 (66.9%)	15.28	School students	Cross-sectional survey design	Watching reality shows negatively affected adolescents’ perceptions of how fairly the police exercise authority. Exposure to the news negatively predicted respondents’ perceptions of the distributive fairness of the police. Perception of procedural justice significantly predicted young people’s attitudes toward the police.
Dirikx and Van Den Bulck (2014)	Belgium	1,968 (51.40%)	16	School students	Cross-sectional survey design	Media use is an antecedent of police cooperation net of the influence of adolescents’ direct police contacts, age, gender, ethnicity, or educational level. When adolescents believe that the police are procedurally fair, they feel an outstanding obligation to obey the police, and they report trust in the police.
Fine et al. (2022)	USA	1,216 males from Crossroads study & 1,169 males from pathways to desistance	15.29 (Crossroads study)	Arrested youths and juvenile offenders	Longitudinal survey design	Among Voice, Neutrality/Impartiality, Distributive Justice/Bias, Respect, and Legitimacy, only Distributive Justice/Bias and Legitimacy were directly associated with concurrent self-reported offending (SRO). All procedural justice scales had indirect effects on subsequent offending through legitimacy.
Flexon et al. (2009)	USA	891 (46%)	16	School students	Cross-sectional survey design	On all four dimensions (priorities, respectfulness, dependability, and competence), commitment to school and seeing other youths stopped and treated disrespectfully by the police were highly significant predictors of trust.
Foster et al. (2022)	USA	2,990 (50–51%)	15	Children from “fragile families”	Longitudinal survey design	Direct and/or vicarious police contact can generate negative attitudes toward the police among African American, Hispanic, and, in certain cases, White youth; however, these effects vary across types of police stop and types of attitude. When a direct stop involved increased officer intrusiveness, African American youth reported reduced respect and increased negative perceptions of procedural justice.
Geller and Fagan (2019)	USA	3,001 (51%)	15.5	Children from “fragile families”	Longitudinal survey design	Both personal and vicarious police contact are associated with increased legal cynicism. Legal cynicism is amplified in teens reporting intrusive contact but diminished among teens reporting experiences characterized by procedural justice.
Harris and Jones (2020)	USA	*N* = 3,427/3,444/2,474 (52.2%)	15.6	Children from “fragile families”	Longitudinal survey design	Direct and vicarious police stops are associated with reduced respect and confidence in the police. The positive effect of police stops on procedural justice is mitigated by police intrusiveness during such stops.
Hinds (2008)	Australia	Study 1: 274 (51%) Study 2: 140 (43%)	Study 1: 15.3 Study 2: 15.7	School students	Longitudinal survey design	Young people who view the police as legitimate are willing to assist the police. Participation in the community policing project had a significant and positive influence on young people’s willingness to assist the police independent of young people’s attitudes toward police legitimacy.
Hofer et al. (2020)	USA	2,406 (54.32%)	15	Children from “fragile families”	Longitudinal survey design (Using data from one single wave)	Relative to youth who experienced only vicarious police contact, youth who had direct or both direct and vicarious police contact reported higher levels of legal cynicism. Youth perceptions of procedural justice were associated with reduced legal cynicism. Situational features of police contact, such as harsh language and frisking, were related to increased legal cynicism.
Jackson et al. (2021)	USA	918 (69.83%)	15	Children from “fragile families”	Longitudinal survey design (Using data from one single wave)	The association between low self-control and diminished perceptions of procedural justice is significantly moderated by officer intrusiveness, i.e., low self-control became increasingly relevant in reduced perceptions of procedural justice as officer intrusiveness decreased.
Jackson et al. (2020)	USA	Vicarious contact: 1,782 (44.05%) Direct contact: 918 (69.83%)	15	Children from “fragile families”	Longitudinal survey design (Using data from one single wave)	Among youth exposed to police stops, the link between low self-control and legal cynicism was robust to perceptions/features of these stops, including the degree of officer intrusiveness, arrest, perceptions of procedural justice, and youth feelings of social stigma following the stop.
Lee et al. (2010)	USA	561 (87.17%)	16	Juvenile offenders	Longitudinal survey design (using data from one single wave)	Youth with a strong sense of ethnic identity perceived increased police discrimination but reported raised positive beliefs regarding police legitimacy. The findings underscore the importance of considering processes that may add salience to legal socialization experiences for adolescents and demonstrate the complex role that ethnic identity plays on discrimination.
Lee et al. (2011)	USA	561 (87.17%)	16	Juvenile offenders	Longitudinal survey design	Increased ethnic identity exploration was related to positive perceptions of police legitimacy and reduced legal cynicism. High ethnic identity affirmation predicted high perceived legitimacy over time, but affirmation was unrelated to legal cynicism after accounting for psychosocial maturity.
Liu and Liu (2018)	China	711 (52%)	16.83	School students	Cross-sectional survey design	Procedural justice and shared values are strong predictors of youth support to the police, and this support positively predicts compliance with the law. Distributive fairness exerts an independent effect on compliance, whereas having been questioned by the police is negatively related to compliance.
McFarland et al. (2019)	USA	3,435 (53.23%)	15.59	Children from “fragile families”	Longitudinal survey design	Participants who reported personal or vicarious police stops had worse self-reported health in adolescence than their counterparts with no contact. Both types of police contact were unrelated to caregiver reports of adolescent health and inconsistently related to somatic symptoms. Procedural injustice exacerbated the relationship between both personal and vicarious contacts and diminished self-reported health. The associations between police contact and self-reported health were stronger among African American and Hispanic adolescents than White ones.
Murray et al. (2021)	UK	2,186 (vary in different models: 1,918/2,110/1,274) The gender ratio not mentioned	12–15	School students	Cross-sectional survey design	Scottish adolescents, who experienced higher volume of stop-and-search, had more negative attitudes toward the police and perceived them to be less procedurally fair than English adolescents.
Nivette et al. (2020)	Switzerland	1,675 (49%)	13, 15, 17, 20 (wave)	School students	Longitudinal survey design	Legal cynicism and police legitimacy decline into early adulthood and exhibit high-rank stability over time. Legal cynicism is closely related to individual characteristics that reflect one’s inability to recognize or abide by internal rules. Police legitimacy is shaped by socialization influences, particularly teacher bonds and police contacts.
Nivette et al. (2022)	Switzerland	232 pairs (55%)	11, 13, 15 (wave)	School students	Longitudinal survey design	If young people feel that they are being treated fairly by their teachers, they are likely to distinguish behaviors that are right or wrong (moral norms), to control their actions (self-control), and to have perceived police legitimacy.
Pehrson et al. (2017)	UK	819 (42.4%)	14–16	School students	Cross-sectional survey design	Perceptions of the police as having goals that align with those of wider society and as being fair in general mediate the relations between the quality of encounters and legitimacy, which in turn mediates the relation with cooperation and compliance. The study finds no support for the distinction between procedural and distributive police fairness as typically conceived.
Pina-Sánchez and Brunton-Smith (2020)	USA	1,354 (Not found)	16	Juvenile offenders	Longitudinal survey design	No directional paths exist between perceptions of police procedural justice and legitimacy while controlling for time-invariant participant heterogeneity. Lagged within-participant perceptions of procedural justice rarely predicted within-participant perceptions of legitimacy or a reciprocal relationship. The study detected substantial time-invariant participant heterogeneity and evidence of legitimacy perceptions being self-reproduced.
Piccirillo et al. (2021)	Brazil	669 (50.71%)	14.5	School students	Longitudinal survey design	Within-person increases in vicarious police contact were linked to decreased police legitimacy over time. The negative effect of vicarious police contact was mediated by evaluations of police procedural justice. The effects of vicarious police contact and violent police contact were mediated by police procedural justice. Additionally, police legitimacy decreased for people who had additional exposure to violence and reduced levels of fear of crime.
Ray (2022)	USA	1,354 (86.4%)	16.04	Juvenile offenders	Longitudinal survey design	Positive personal and vicarious experiences with the police had positive effects on legal orientations (i.e., legitimacy and cynicism), whereas vicarious experiences were more influential on offending than personal experiences. These effects were consistent across race/ethnicity. Both changes in legitimacy (positive) and cynicism (negative) were significant for understanding the differences in offending; however, the effect of cynicism was more consistent across race/ethnicity than that of legitimacy. Procedurally just treatment of juveniles by the police can enhance legal compliance.
Reisig and Lloyd (2009)	Jamaica	289 (41%)	Not controlled in the model	School students	Cross-sectional survey design	The correlation between procedural justice judgments and police legitimacy is positive and statistically significant. Students who favorably rate police practices in terms of procedural justice also report a willingness to help the police fight crime.
Rodrigues and Medina (2021)	Brazil	800, 743, 724 different waves (50%)	11, 12, 13 (wave)	School students	Longitudinal survey design	Student perception of teacher legitimacy is a consistent predictor of police legitimacy. Students who view their teachers as procedurally just are also likely to consider the police as procedurally just.
Schwarzenbach et al. (2020)	Germany	2,921 (54.3%)	~15	School students	Cross-sectional survey design	Negative attitudes toward the police (including police legitimacy) strongly relate to young people’s intentions to retaliate. Young people who associate with delinquent peers and engage in unsupervised activities tend to have retaliatory conduct.
Shook et al. (2021)	USA	227 (56%)	16.2	Juvenile offenders	Cross-sectional survey design	Youth who feel they were treated fairly by their defense attorneys viewed the police and courts as legitimate. White youth viewed the police as more legitimate than the youth of color did; girls viewed courts as less legitimate than boys did.
Sindall et al. (2017)	UK	1,657 (52.1%)	12–13	General population	Cross-sectional survey design	Young people whose parents have positive views of the police tend to hold positive views. Experience of victimization is linked to pessimistic assessments of the police. Children from married households report positive views of the police. Young people’s confidence in the police declines as they grow older.
Slocum et al. (2016)	USA	2,919 (49.6%) 4 waves	11.83	School students	Longitudinal survey design	Police contact amplifies delinquency by increasing feelings of procedural injustice and in turn support for the personal use of violence.
Slocum and Wiley (2018)	USA	3,245 (50.2%)	11.85	School students	Longitudinal survey design	For several outcomes (procedural injustice, willingness to report, and delinquency), all youth were protected from the potentially negative consequences of police encounters when they were satisfied with their treatment.
Thompson and Pickett (2021)	USA	1,354 (86%)	16.04	Juvenile offenders	Longitudinal survey design	Individuals who experience positive encounters with the police tend to grant them great legitimacy. Negative police encounters are more strongly associated than positive encounters with legitimacy. Police legitimacy was negatively associated with offending.
Thompson and Wilson (2022)	USA	330 (90%)	15.94	Juvenile offenders (Mexican Americans)	Longitudinal survey design (using data of a subsample)	Changes in procedural justice perceptions are significantly related to changes in Mexican identification, whereas procedural justice is unrelated to changes in Anglo identification.
Trinkner et al. (2020)	Brazil	743 (50%)	12	School students	Longitudinal survey design (using data from one single wave)	Both procedural justice and crime perceptions were associated with higher perceptions of the police as legitimate authority figures. Procedural justice was not associated with legal cynicism, although crime perceptions were positively associated. Police legitimacy was associated with substantially reduced odds of offending. Legal cynicism had no relation with offending.
Walsh et al. (2019)	USA	1,216 (100%)	15.29	Male juvenile offenders	Longitudinal survey design	Positive experiences with the police following the youth’s first arrest were associated with less self-reported delinquency 2 years later, which was partially mediated by reductions in adolescents’ cynicism toward the legal system.
Trinkner et al. (2019)	USA	308–393 (30.3–39.4%) Four waves	Four waves: 15.17–17.9	School students	Longitudinal survey design Experimental study with the use of vignette case	Both experimental manipulations led to increased perceptions of situational procedural justice and officer legitimacy. Prior perceptions of police legitimacy did not predict judgments of situational procedural justice; however, in certain cases, previous engagement in delinquency was negatively related to situational procedural justice. Prior perceptions of legitimacy were positively associated with situational perceptions of legitimacy regardless of experimental manipulations.

**Table 2:** Qualitative studies (including individual interviews, focus group interviews, and observation methods) (*n* = 9).

	Country	*N* (% males)	Mean age	Sample	Methods	Relevant findings
Adorjan et al. (2017)	Canada	21 (7 males+13 females + 1 no gender)	15–17	Youth from rural communities	Focus group interview	Trust and a sense of police fairness is instilled only when the police are seen to be “on the same side” as the citizens being policed. The impact of vicarious experience on the youth’s personal views of the police was highlighted.
Brunson and Miller (2006)	USA	40 (100%)	16	African-American youths from an urban community	Survey and in-depth interviews	African-Americans—both adult and juvenile—report more dissatisfaction with and distrust of the police than other groups. African Americans disproportionately report “getting hassled” by the police and disproportionately experience a range of additional negative police actions.
Clayman and Skinns (2012)	UK	24 (gender is unspecified)	13–16	School students	In-depth interviews	Peers, specifically “older ones,” appear to have more influence on a young person’s decision to cooperate with the police (snitching) than their relationships with the police or the advice from their family.
Dirikx et al. (2012)	Belgium	106 (50%)	13–19	School students	Focus group interview	Vicarious and mediated contacts with unfair police behavior were also found to have an influence but did not seem to leave as lasting an impression as that left by direct experiences. In the low-crime areas, police officers were found friendly, trustworthy, and neutral. In the high-crime neighborhoods, police officers were regarded as acting disrespectfully.
Gleeson (2018)	UK	20 (16 males)	12–18	Youth from youth centers	In-depth interviews	Attitudes toward the police were influenced by a range of factors, including, feeling stereotyped by the police, lacking control during interactions, and a lack of voice in dealing with the police.
Hough (2013)	UK	49 officers +32 youth	Under 18	Young people from different ethnic groups	Observation and in-depth interviews	The professionalized style of policing instead of procedural justice is related to police legitimacy.
Pennington and Farrell (2019)	USA	30 juveniles (23 males)	NA	Youth and families in juvenile justice systems	Observation and semi-structured interviews	Voice was a process of negotiating dialogue between court officials and court participants throughout the legal process. The police should be held accountable in courtrooms.
Ricciardelli et al. (2020).	Canada	59 (32 males)	15	School students	Semi-structured focus group	Rural youths offered complex and sometimes contradictory views of the police. Maintaining trust is difficult when strong personal relations exist with the police.
Saarikkomäki (2016)	Finland	31			Focus group interview	Authorities practice fair and unfair treatments: (1) Fair treatment consists of peaceful and predictable interactions and mutual respect; (2) Unfair narratives consisted of impolite and aggressive treatment.

### 2.4 Charting the data (data extraction)

The relevant information was extracted from the selected publications, including author names, year of publication, location of the study, sample size and percentage of male participants, the mean age of the sample, types of the sample (e.g., general populations, school students, or offenders), research methods (study design for empirical studies or secondary data analysis), and main results. The studies were categorized into four groups (see Tables 1, 2).

## 3 Results

The fifth and final stages of Arksey and O’Malley’s (2005) scoping review framework encompasses summarizing and reporting the findings. All 47 articles in our sample were empirical studies. The article characteristics are summarized in Table 1 (quantitative studies = *38*) and Table 2 (qualitative studies = *9*). All these articles were published between 2006 and 2022, and most (74.47%, *n* = *35*) were published after 2015. More than half of the publications came from North America (*n* = *26*), including the United States (*n* = *24*) and Canada (*n* = *2*). Other publications were from European countries, including the United Kingdom (*n* = *6*), Belgium (*n* = 3), Switzerland (*n* = *2*), Finland (*n* = *1*), Germany (*n* = *1*), and Spain (*n* = *1*). The remaining publications were from Latin America (Brazil, *n* = *3*; Jamaica, *n* = *1*), Asia (China, *n* = *1*), Africa (Nigeria, *n* = *1*), and Australia (*n* = *1*). There were 53,562 participants across the 47 studies (see Tables 1, 2). Specifically, the samples in the quantitative studies ranged from 140 to 3,435. The percentage of males varied from 34.5 to 100%. The sample sizes in the qualitative studies (Table 2) ranged from 20 to 106. This review answered the following questions related to police legitimacy and procedural justice.

### 3.1 How are police legitimacy and procedural justice defined and measured in the literature?

Over the decades, police legitimacy has been defined or interpreted by legal, philosophy, and social sciences scholars. Beetham (1991, p. 16) argued that legitimacy can only exist when “it conforms to established rules, the rules can be justified by references to beliefs shared by both dominant and subordinate, and there is evidence of consent by the subordinate.” Tyler (2006, p. 376) defined legitimacy as “belief that authorities, institutions, and social arrangements are appropriate, proper, and just.” Bottoms and Tankebe’s (2012, p. 164) definition of legitimacy involves a “recognition of the moral rightness of claims to exercise power here and now, rather than in the future.” Murphy (2017, p. 45) put it: “Legitimacy reflects the degree to which people recognize the right of an authority to govern their behavior.” Clearly, as Cao (2022, p. 9) mentioned, the police legitimacy is “multidimensional,” and its concept is “quite liquid.”

The most common definition of procedural justice was the one used in Sunshine and Tyler (2003), Tyler (1990), and Tyler and Huo (2002), which emphasizes people’s perception or judgment of the fairness of procedures and treatment. Another definition of procedural justice was respectful, impartial, and unbiased treatment and voice (see Fagan and Tyler, 2005; Hamm et al., 2017; Jackson et al., 2013; Trinkner and Tyler, 2016). The third definition of procedural justice emphasized police officers’ effectiveness and distributive fairness (Tankebe, 2013) and their position as an appropriate authority entitled to obedience (Tyler, 2006).

Notably, although most scholars defined and measured the concepts of legitimacy and procedural justice separately (e.g., Fine et al., 2022), some scholars combined the two concepts into one by including procedural justice in the construct of legitimacy. For example, Nivette et al. (2020, pp. 71–72) concluded from their literature review that police legitimacy “is often used to capture broader perceptions of police performance, including procedural justice, lawfulness, trust, effectiveness, distributive justice, and obligation to obey the police.” Another example is the definition used by Liu and Liu (2018), which included procedural fairness and distributive fairness as two dimensions of legitimacy. The association between procedural justice and legitimacy was empirically tested in several studies. Among the 38 quantitative studies in this review, 11 specifically identified a significant positive association between procedural justice and police legitimacy. One exception was a longitudinal study (Pina-Sánchez and Brunton-Smith, 2020) that found no connection between perceptions of police procedural justice and legitimacy.

#### 3.1.1 Definitions (measurements) in quantitative studies

As shown in Table 3, among the 38 cross-sectional, longitudinal, and experimental studies, the number of items used to measure legitimacy ranged from 1 (Reisig and Lloyd, 2009) to 12 (Akinlabi, 2017), and Cronbach’s *α* varied from 0.67 (Hinds, 2008) to 0.87 (Liu and Liu, 2018; Nivette et al., 2020; Ray, 2022). Typical items include “I have a great deal of respect for the police,” “Overall, the police are honest,” and “I feel people should support the police” (Fine et al., 2022). In another study, some more items regarding obligation to obey like “People should do what the police tell them even when they do not like the way police treat them,” “People should do what the police tell them to do, even when they do not understand why the order has been given” and “People should do what the police tell them to do, even when they disagree with the police order” were used (Akinlabi, 2017). The number of items used to measure procedural justice varied from 3 (Baz and Fernandez-Molina, 2018; Foster et al., 2022; Geller and Fagan, 2019; Harris and Jones, 2020; Jackson et al., 2021; Jackson et al., 2020; Liu and Liu, 2018; McFarland et al., 2019; Slocum and Wiley, 2018) to 19 (Lee et al., 2010; Lee et al., 2011; Pina-Sánchez and Brunton-Smith, 2020; Ray, 2022), and Cronbach’s α ranged from 0.57 (Lee et al., 2010; Ray, 2022) to 0.88 (Shook et al., 2021). Sampled items include “Police take account of the needs and concerns of people they deal with” (Akinlabi, 2017), “whether the police gave the opportunity for others to express their side of the story” and “whether the police talked politely” (Rodrigues and Medina, 2021).

**Table 3:** Main measures used to address police legitimacy and procedural justice in studies related to young people (*n* = 38).

	References	Number of items	Internal consistency	Number of items	Internal consistency
		(Police legitimacy)	(Cronbach’s *α*)	(Procedural Justice)	(Cronbach’s α)
1	Akinlabi (2017)	12	0.86	12	0.78
2	Baz and Fernandez-Molina (2018)	4	0.81	3	0.8
3	Cavanagh and Cauffman (2019)	0	0.79–0.83	0	0
4	Cohn et al. (2012)	10	0.83	0	0
5	Dirikx et al. (2013)	0	0	4	0
6	Dirikx and Van Den Bulck (2014)	5	0.85	7	0.84
7	Fine et al. (2022)	4	0	13	0
8	Flexon et al. (2009)	0	0	0	0
9	Foster et al. (2022)	0	0	3	0
10	Geller and Fagan (2019)	0	0	3	0.71
11	Harris and Jones (2020)	0	0	3	0.72
12	Hinds (2008)	4	0.67&0.71	4	0.7&0.74
13	Hofer et al. (2020)	0	0	0	0
14	Jackson et al. (2021)	0	0	3	0.77
15	Jackson et al. (2020)	0	0	3	0.81
16	Lee et al. (2010)	6	0.77	19	0.57&0.74
17	Lee et al. (2011)	6	0.77	19	0.74
18	Liu and Liu (2018)	6	0.87	3	0
19	McFarland et al. (2019)	0	0	3	0.7
20	Murray et al. (2021)	5	0	0	0
21	Nivette et al. (2020)	3	0.82–0.87	0	0
22	Nivette et al. (2022)	3	0.81	0	0
23	Pehrson et al. (2017)	7	0.73	0	0
24	Pina-Sánchez and Brunton-Smith (2020)	11	0	19	0
25	Piccirillo et al. (2021)	6	0.77	4	0.84
26	Ray (2022)	11	0.8–0.87	19	0.57–0.79
27	Reisig and Lloyd (2009)	1	0	6	0.71
28	Rodrigues and Medina (2021)	3	0.71	4	0.86
29	Schwarzenbach et al. (2020)	4	0.73	0	0
30	Shook et al. (2021)	6	0.78	5	0.88
31	Sindall et al. (2017)	0	0	0	0
32	Slocum et al. (2016)	0	0	0	0
33	Slocum and Wiley (2018)	0	0	3	0.85
34	Thompson and Pickett (2021)	6	0.833	16	0.771&0.645
35	Thompson and Wilson (2022)	0	0	4	0.76
36	Trinkner et al. (2020)	5	0.71	4	0.75
37	Walsh et al. (2019)	5	0.68	0	0
38	Trinkner et al. (2019)	10	0	13	0

#### 3.1.2 Definitions (meanings) in qualitative studies

This review unpacked the meaning of these terms to the research targets (i.e., children, adolescents and young people). Several qualitative studies described young people’s subjective definitions/descriptions of police legitimacy or procedural justice as follows: being “helpful,” “protective,” and “not resorting to aggression and violence” (Gleeson, 2018, p. 103); being “sincere in their presentations of self and the situation” (Adorjan et al., 2017, p. 566); “speak[ing] to young people by coming into schools and youth clubs” (Clayman and Skinns, 2012, p. 469); “being held accountable in the courtrooms” (Pennington and Farrell, 2019, p. 361); “friendliness” and “openness” and “accessibility and trust” (Ricciardelli et al., 2020, pp. 204–208); “peaceful, friendly and respectful interactions” with the police (Saarikkomäki, 2016, p. 1261); and “fairness—polite and equal treatment of diverse groups” (Dirikx et al., 2012, pp. 199–201). Clearly, some of the responses in these qualitative studies aligned with the measurement items in the survey studies, such as trust and fairness, whereas some of the qualitative interpretations of police legitimacy/procedural justice appeared only in qualitative studies, such as friendliness and openness. In addition, the qualitative responses generated by the young participants created a picture of how procedural justice can be put in place in a specific and contextually relevant way. For instance, quantitative studies do not specify how young people develop their subjective definitions of police procedural justice. However, a focus group study conducted in a rural area of Canada (Adorjan et al., 2017, p. 562) included a youth who described the process of shaping his definition: *“My parents watch a lot of the cop shows like CSI and stuff like that and a lot of movies got cops in them. But a lot of the movies that got cops in them make the cops look like the bad guys and the good guys look like or the bad guys look like the good guys right. But other than that I do not think there would be anywhere else where I would get an idea from.”* Additionally, a study based on 24 in-depth interviews with school students in the UK illustrated how pop music and youth-gang culture promoted an anti-snitch mentality and a code of silence among peers (Clayman and Skinns, 2012). Clearly, qualitative studies provide a more contextualized understanding of the formation of police legitimacy and procedural justice compared to quantitative research.

### 3.2 What are the determinants of police legitimacy among children and youth?

#### 3.2.1 Procedural justice, police behavior, and policing style (*n* = 14)

Among the correlates of police legitimacy, police procedural justice was the most frequently studied factor. Procedural justice was found to be positively associated with police legitimacy and negatively associated with legal cynicism (Akinlabi, 2017; Baz and Fernandez-Molina, 2018; Fine et al., 2022; Geller and Fagan, 2019; Hofer et al., 2020; Jackson et al., 2020; Trinkner et al., 2020; Ray, 2022; Reisig and Lloyd, 2009), with one exception (Pina-Sánchez and Brunton-Smith, 2020). Some studies demonstrated the impact of one or more elements of police procedural justice on police legitimacy. For example, police goal alignment with the goals of the broader society was found to mediate the relationship between quality encounters and legitimacy (Pehrson et al., 2017) and instilled “a sense of police fairness” (Adorjan et al., 2017, p. 566). The youth’s perception of police corruption (Akinlabi, 2017) was negatively related to the perceived legitimacy of the police. This is understandable, as fairness can hardly be achieved in a society with police corruption. One qualitative study focused on a key element of procedural justice (i.e., voice) to understand how having or not having a voice affects legitimacy (Pennington and Farrell, 2019). Another qualitative study emphasized that a respectful, polite, empathetic, and peaceful manner of police officers increased young people’s perceptions of the legitimacy of legal authorities, including police officers (Saarikkomäki, 2016).

#### 3.2.2 Experience/contact with the police (*n* = 10)

Both the quantitative and qualitative studies in this review supported the relationship between perceived police legitimacy and quality of contact. For example, a survey study of students found a significant positive relationship between police contact and perceived police legitimacy (Nivette et al., 2020). Positive personal and vicarious experiences with the police positively affected legitimacy (Ray, 2022). Repeated exposure to law enforcement officials in a positive, nonenforcement capacity may improve young people’s legitimacy perceptions (Fine et al., 2022). Similarly, officer intrusiveness in a direct stop was found to be negatively associated with youth perception of procedural justice for people of African American, Hispanic, and White youth (Foster et al., 2022). Another study demonstrated that legal cynicism is amplified by intrusive contact and diminished by contact with procedural justice, as reported by teens (Geller and Fagan, 2019). Youths with few encounters with the police were likely to ascribe greater legitimacy to the police than those with many encounters (Akinlabi, 2017). Contact with the police due to the commission of crimes reduced perceived police legitimacy (Baz and Fernandez-Molina, 2018). One qualitative study found that adolescents’ less favorable views were related to negative police contacts (Dirikx et al., 2012). Another qualitative study highlighted the impact of vicarious experiences on the adolescents’ personal opinions of police (Adorjan et al., 2017). A qualitative study concluded that a policing style with the components of interventionism (i.e., readiness to intervene) and professionalism (i.e., adherence to the principles of procedural justice) could help secure young people’s compliance with the law (Hough, 2013).

#### 3.2.3 Relationship with other authority figures (*n* = 8)

School and personnel situations were found to be related to police legitimacy in many studies. Perception of police legitimacy was influenced by school attachment (Baz and Fernandez-Molina, 2018). Commitment to school was a significant predictor of police trustworthiness (Flexon et al., 2009). Police legitimacy was found to be shaped by teacher bonds (Nivette et al., 2020), perceived fair treatment by teachers (Nivette et al., 2022), and parental monitoring/attitudes (Baz and Fernandez-Molina, 2018). Lawyer procedural justice (Shook et al., 2021), teacher procedural justice (Rodrigues and Medina, 2021), and teacher legitimacy (Rodrigues and Medina, 2021) were found to be linked to young people’s perceptions of police legitimacy.

#### 3.2.4 Legal and moral reasoning, self-justice retaliation, self-control, and offending (*n* = 8)

Individual circumstances were connected to respondents’ views of police legitimacy in many studies. Legal reasoning (Cohn et al., 2012), moral neutralization (Akinlabi, 2017; Nivette et al., 2020), cynicism (Nivette et al., 2020), self-justice retaliation (Schwarzenbach et al., 2020), and offending/deviant behaviors/delinquency (Akinlabi, 2017; Nivette et al., 2020) were associated with perceived police legitimacy. The indirect influence of low self-control on police legitimacy was also evident (Nivette et al., 2022).

#### 3.2.5 Neighborhood (*n* = 3)

Neighborhood crime perceptions were positively related to legal cynicism (Trinkner et al., 2020). One qualitative study found that youth perceptions of the police varied between high-and low-crime neighborhoods: “in the low-crime neighbourhoods the youths were more positive stating that the police have their flaws but often do a good job” (Dirikx et al., 2012, p. 199). Another qualitative study indicated that young people trusted the police in small towns where the police are prominent and informally accessible (Ricciardelli et al., 2020).

#### 3.2.6 Social media (*n* = 2)

Social media also plays a role in shaping young people’s perceptions of police legitimacy. A cross-sectional survey of 356 Flemish adolescents found that watching police reality shows and exposure to the news negatively predicted respondents’ perceptions of the distributive fairness of police procedures (Dirikx et al., 2013). Another study demonstrated that the youths’ procedural fairness judgment of the police was negatively predicted by exposure to commercial channel crime shows but positively predicted by exposure to public channel crime shows in Belgium (Dirikx and Van Den Bulck, 2014).

### 3.3 Who are the vulnerable groups receiving less legitimate and less just police treatment?

Demographic differences in gender, ethnicity (race), age, and household structure were controlled for and detected in most of the reviewed quantitative studies. However, the qualitative studies did not focus on demographic factors. Some studies discussed the impact of demographic and individual characteristics on police legitimacy.

#### 3.3.1 Age (*n* = 12)

Age was generally negatively associated with police legitimacy. Older participants were less likely to perceive the police as legitimate (Cohn et al., 2012; Fine et al., 2022; Lee et al., 2010; Trinkner et al., 2019). In addition, age was also positively correlated with decreases in or lack of confidence in the police (Foster et al., 2022; Harris and Jones, 2020; Sindall et al., 2017) and lower levels of respect for and confidence in the police (Harris and Jones, 2020). Legal cynicism increased with respondent age (Jackson et al., 2020). Willingness to assist the police was negatively predicted by age (Hinds, 2008). In a longitudinal study conducted in Brazil between 2016 and 2018, more positive views of police fairness were linked to increased parental involvement at T1 (aged 11) but not at T2 (aged 12) and T3 (aged 13) as students grew older (Rodrigues and Medina, 2021).

#### 3.3.2 Race (*n* = 10)

Ethnic minorities (e.g., African Americans and Latinos) are less likely to view the police as legitimate (Fine et al., 2022; Foster et al., 2022), have less respect for the police (Harris and Jones, 2020), show less confidence in the police (Harris and Jones, 2020), and have a higher level of legal cynicism (Hofer et al., 2020; Geller and Fagan, 2019; Ray, 2022) than people who are not ethnic minorities. A person with the minority status is unlikely to display support for the police (Liu and Liu, 2018). One qualitative study concluded that African Americans (both adults and juveniles) reported more dissatisfaction and distrust of the police (Brunson and Miller, 2006) than other Americans. Nevertheless, the perception that minority status is negatively related to perceived police legitimacy is not constant. For example, based on the first wave of data from a longitudinal study of young African American offenders, youths with a strong sense of ethnic identity perceived more police discrimination but reported more positive beliefs regarding police legitimacy (Lee et al., 2010) than those with a weak sense of ethnic identity. The longitudinal data indicated that increased ethnic identity exploration was related to positive perceptions of police legitimacy (Lee et al., 2011).

#### 3.3.3 Gender (*n* = 8)

Regarding children’s and young people’s perceptions of police legitimacy and gender, the selected studies gave mixed conclusions. Many studies found that female respondents were more likely than male respondents to adopt a negative view of police legitimacy (Baz and Fernandez-Molina, 2018; Piccirillo et al., 2021). Another study found a similar pattern in African American but not in White and Latino participants (Ray, 2022). The opposite conclusion was reached in other studies, with female participants reporting higher levels of police legitimacy, compliance, and confidence than male participants (Pehrson et al., 2017; Sindall et al., 2017). In addition, male participants expressed higher legal cynicism (Hofer et al., 2020) and more police discrimination (Lee et al., 2010) than female participants. Boys in a longitudinal study showed a significant increase in belief in police legitimacy between adolescence and adulthood (Nivette et al., 2020).

#### 3.3.4 Household structure (*n* = 1)

One study showed that children from married households, compared with single-parent households, reported more positive views of the police (Sindall et al., 2017).

#### 3.3.5 Other disadvantageous conditions (*n* = 5)

In addition to demographic characteristics, individual circumstances were related to respondents’ perceptions of police legitimacy. Offenders, criminals, participants with delinquent behaviors had significantly lower perceptions of legitimacy than participants with no criminal records (Nivette et al., 2020). People with low self-control (Nivette et al., 2022) and little internal moral control (Akinlabi, 2017; Cohn et al., 2012; Nivette et al., 2020) had unfavorable perceptions of police legitimacy.

### 3.4 What are the consequences of procedural justice for police legitimacy?

#### 3.4.1 Compliance (*n* = 9)

Some studies revealed that police legitimacy was negatively related to delinquent actions/rule-violating behavior/offending (Baz and Fernandez-Molina, 2018; Slocum et al., 2016; Slocum and Wiley, 2018; Thompson and Pickett, 2021; Trinkner et al., 2020; Walsh et al., 2019) and positively related to compliance with the law (Liu and Liu, 2018; Murray et al., 2021) and obligation to obey (Dirikx and Van Den Bulck, 2014).

#### 3.4.2 Cooperation and willingness to assist (*n* = 5)

Some studies demonstrated that police procedural justice and legitimacy were positively related to intentions to cooperate (Dirikx and Van Den Bulck, 2014; Pehrson et al., 2017) and willingness to help police fight crime (Hinds, 2008; Reisig and Lloyd, 2009). Nevertheless, one qualitative study concluded that underaged persons’ decision to cooperate with the police was more strongly impacted by broader social contexts (e.g., their safety, peer groups, gangs, and families) than by police procedural justice (Clayman and Skinns, 2012).

#### 3.4.3 Trust (*n* = 5)

Trust in police was found to be an outcome of procedural justice (Dirikx and Van Den Bulck, 2014). Students who had observed other youths being stopped and treated disrespectfully by the police (procedural injustice) expressed significantly less trust in the police than those who had not observed such interactions (Flexon et al., 2009). The relationship between trust and police procedural justice was unpacked in qualitative studies. For example, trust and a sense of police fairness were present only when the police were seen “on the same side as the citizens being policed” (Adorjan et al., 2017, p. 566). African Americans, both adults and juveniles, reported high levels of dissatisfaction and distrust of the police (Brunson and Miller, 2006). A focus group of 60 youth from a Canadian rural area concluded that maintaining trust is more difficult when strong personal relations with the police exist because “youth appear to question the extent of their confidentiality if they were to consider making a police report or requesting help” (Ricciardelli et al., 2020, p. 208).

#### 3.4.4 Cynicism (*n* = 4)

In a survey of juvenile offenders, increases in police legitimacy were associated with decreases in cynicism (i.e., a lack of empathy, defiance, impatience, and disregard for authority) (Ray, 2022). Some studies revealed that legal cynicism resulted from perceived lack of or diminishing procedural justice/fairness or legitimacy (Geller and Fagan, 2019; Jackson et al., 2020). Youth perceptions of high procedural justice were associated with low legal cynicism (Hofer et al., 2020).

#### 3.4.5 Propensity to retaliate (*n* = 1)

Drawing on survey data from 2,921 young people in two German cities, one study found that the propensity to engage in retaliatory actions was positively related to police-initiated contact (Schwarzenbach et al., 2020).

#### 3.4.6 Health (*n* = 1)

A longitudinal study revealed that police procedural injustice exacerbated the relationship between personal and vicarious contact and diminished self-reported physical health (McFarland et al., 2019). In McFarland and his team’s elaboration, contact with police could be a health-salient stressor detrimental to youth development.

## 4 Discussion

This study summarizes research on perceived police legitimacy and procedural justice among youth. It is one of the first reviews to synthesize and discuss the definitions, determinants/correlates, and consequences of police legitimacy relevant to young people based on qualitative and qualitative studies. We summarize the main findings below.

### 4.1 Perspective and measurement issues

Both quantitative and qualitative researchers contributed to conceptualizing and operationalizing police legitimacy. Among the 38 quantitative papers in this review, procedural justice, lawfulness, trust, effectiveness, distributive justice, obligation to obey the police, process fairness, and treatment fairness were used to measure police legitimacy. The terms mentioned in the previous sentence refers to the quality of treatment in police-youth encounters. Research focuses on how well the young people are handled by the police—involving a one-way, give-and-take relationship. Among the nine qualitative papers, more detailed and subjective conceptualizations were presented: friendliness, openness, accessibility, trust, peaceful and respectful interaction, and speaking to children and youth in schools and youth clubs. These are not only quality of treatment but also involve the interaction processing (or communication) and assessment of fulfilling personal expectations—involving a two-way, reciprocal relationship. We echo Bottoms and Tankebe’s (2012) argument that legitimacy is a dialogic process between power-holders (e.g., the police) and their audience (e.g., children and youth). Seemingly, a holistic perspective to understand the *quality of treatment and interaction in encounters* is imperative.

It is worth noting that 45 of the 47 studies were conducted in Western contexts; the exceptions were one from China and one from Africa. Whether the theoretical and conceptual understanding of police legitimacy can be applied to non-Western societies remains unclear. Cross-cultural comparisons and qualitative, in-depth, and contextual understandings of the definitions are warranted. Contextual understanding is essential, especially since we need to differentiate if people obey the law due to “true legitimacy”—their moral obligation to obey or “dull compulsion”—compliance is a result of “structurally-generated apathy,” “pragmatic acquiescence” “*de facto* authority” (Bottoms and Tankebe, 2012, pp. 148 and 165). Dull compulsion is more possible in those societies under authoritarian governance. We need to interpret the research data about people’s obligation to obey cautiously.

In the studies reviewed, the measurements of “global procedural justice” and “specific procedural justice” are not differentiated precisely. For instance, respondents were generally asked about the extent to which they perceived fairness and respect from the police. According to Gau (2014, p. 190), the former refers to “civilians’ general assessments of the overall levels of procedural justice that police provide to the public during typical face-to-face encounters” (p. 190), while the latter pertains to “evaluations made by individuals who have experienced face-to-face interactions with officers and are in a competent position to judge the extent to which officers acted with respect, fairness, and impartiality.” Essentially, the assessment of global procedural justice is not necessarily derived from firsthand experience; instead, media and vicarious experiences are major sources of judgment. Additionally, in many studies, while reporting factor loadings and Cronbach’s alphas is helpful, it is limited because the discriminant and convergent validity of these measurements have yet to be established (Gau, 2014). We endorse Gau’s perspective, advocating for confirmatory factor analysis to elucidate the underlying factor structure. Subsequently, employing structural equation models becomes imperative to discern the predictive interrelationships among these factors in quantitative inquiries.

### 4.2 Implications for practices and services

The review supports a policing style characterized by fairness and justice and offers insights into promoting police legitimacy and mitigating the negative consequences of inadequate legitimacy.

First, from a crime control and prevention perspective, police legitimacy is valuable, as compliance with the law, obligation to obey, cooperation with and willingness to offer assistance to the police, and trust were associated with police legitimacy in nearly half of the studies. From the perspective of advancing children’s and youths’ well-being, police legitimacy is indispensable. Some studies demonstrated that propensity to retaliation, cynicism, and mental health issues are connected to diminished and inadequate perceptions of police legitimacy. Therefore, the need for police legitimacy and procedural justice is strongly justified by this review, not only because they improve society but also for the sake of citizens in general and minors in particular. Nevertheless, some scholars (e.g., Bottoms and Tankebe, 2012; Cao, 2022) reminded us of dull compulsion (i.e., people feel compelled to obey the order of authority or to obey the law for reasons other than legitimacy). We need to be aware of the differences in social and political contexts when interpreting the association between police legitimacy and compliance.

Second, the means used to achieve police legitimacy is a crucial question. Our findings indicate that establishing police legitimacy depends on several related and independent factors. These factors range from demographics (e.g., gender, age, and race/ethnicity) to personal qualities of youths (e.g., individuals’ legal and moral reasoning, self-justice retaliation, and self-control). Additionally, organizational and officer factors (e.g., police behavior, police contacts, and the relationships of youth with other authority figures) also matter. The social support system (e.g., parents, teachers, and legal practitioners), neighborhood circumstances (e.g., income level, and crime-rate), and societal influence (e.g., social media) also contribute significantly to the establishment of police legitimacy. Therefore, building and maintaining police legitimacy requires the intervention of multiple parties. For police officers, “the vital first step to building police legitimacy is to improve procedurally just policing practices through training, retraining, and oversight” (Fine et al., 2021, p. 15). Police leaders may need to actualize internal procedural justice (e.g., fair and just treatment of officers) so that supervisors and frontline officers can learn and apply it when dealing with the public. Indeed, internal and external procedural justice systems were shown to be connected in several studies, with officers who received supervisory procedural justice within an organization being more likely to render fair treatment to the public (e.g., Sun et al., 2018; van Craen and Skogan, 2017). Training in communicating with adolescents and ethnic minority groups, including skills such as active listening, empathic understanding, and rapport building with adequate cultural sensitivity, is essential for fair and quality policing. As children’s and youth’s relationships and interactions with their parents and teachers can impact their perceptions of police legitimacy, adults with a guardian role should be encouraged to instill proper legal and moral reasoning in the younger generation and to establish bonds with them. For example, Fine et al. (2021) concluded from a longitudinal study of 1,200 male adolescent offenders that parent training and family intervention are vital treatments for children and youth, demonstrating the possibility of intergenerational transmission of legal values and beliefs. For children and youth, assistance can be offered to enhance their moral reasoning and self-control abilities and to challenge tendencies to moral neutralization and self-justice retaliation. For example, Sheeran et al. (2022) mentioned a program entitled Students Talking it Over with the Police (STOP) specifically targeted youth. This program consisted of a standardized curriculum facilitated by two officers and taught to roughly 10 to 12 youths. The results indicated that participating in the STOP group significantly increased positive perceptions of the police. For this type of intervention, the collaborative effort between police officers and helping professionals, such as counselors and social workers, is critical. However, program providers need to bear in mind that the ultimate objective of those training programs is to promote “true legitimacy (i.e., compliance gained due to a perceived moral obligation to obey), not “dull compulsion” (compliance gained out of pragmatism or a result of *de facto* authority) (Bottoms and Tankebe, 2012, p. 165). Last, community leaders, especially those in high-crime neighborhoods, can mobilize local resources (e.g., venues, money, and volunteer workforces) to create opportunities for informal/positive contact between police officers and youngsters. In addition, communities can create drop-in centers staffed by social workers, mental health professionals, and other service providers (Slocum and Wiley, 2018). These collective efforts to restore social order can be helpful, as youth who experience less informal forms of social control in their neighborhoods view the police as less legitimate (Shook et al., 2021). However, such community initiatives rarely succeed without support from the government, especially in economically disadvantaged communities.

Third, the following problem deserves our efforts: determining a remedy for the harm to minors caused by a lack of procedural justice during contact (e.g., stops and searches) with the police. Such harm includes cynicism (i.e., a lack of empathy, defiance, impatience, and disregard for authority), the propensity to adopt self-justice retaliation (e.g., solving the problem through violence instead of calling the police), and adverse health consequences. For this reason, there is a high demand for the provision of services to arrested youth and children who have frequent police-initiated contacts on the street (e.g., delinquent youth). These vulnerable groups may need social workers to reach out and assist them in managing the anxiety, anger, and shame resulting from negative police encounters. Additionally, engaging underaged in a restorative justice program could be considered. In such a program, youths could voice their grievances about the perceived injustice, which might reduce their tendency to develop cynicism toward the police and government.

### 4.3 Implications for research advancement

This review identified some knowledge and methodological gaps that can be filled by future research. First, most of the papers in this review used quantitative survey-based methods. Despite their empirical contribution, such quantitative methods cannot fill all the knowledge gaps, such as the effectiveness of police procedural justice practices in enhancing children’s and youth’s perceptions of police legitimacy. Evidence-based research using experimental designs can help to fill this gap. Some policing studies have adopted experimental designs, such as using a randomized controlled trial to evaluate the effectiveness of procedural justice training for officers (Antrobus et al., 2019), short procedurally just traffic encounters with the police (Mazerolle et al., 2013), or a factorial vignette design (Reisig et al., 2018), but such studies are rare. Second, procedural justice is process-based. Understanding the dynamics and interactions of the process is essential, and qualitative methods capture these elements more effectively than quantitative methods through the interviewees’ narratives. Accordingly, researchers must first be sensitive to the power and research ethics issues involved in such methods, such as confidentiality and gaining informed consent in the data collection process from vulnerable groups, including children and youth.

Third, as perceptions and judgments of police behavior are situational, methods that use scenarios and vignette cases may help to unpack the complexity of these concepts. For example, Trinkner et al. (2019) conducted a vignette-based experiment that manipulated two aspects of officer behavior linked to the perceptions of police fairness: voice and impartiality. The application of vignettes in this study helped confirm that both the voice and impartiality manipulations impacted judgments of situational procedural justice. The use of vignettes in this field of study should be encouraged. Indeed, diversification methodologies to understand perspectives in the research of police legitimacy may help to unpack the theoretical model with reference to the context of policing (Graham, 2022). Fourth, as proposed by Sun and Wu (2022), further studies should be conducted to examine other consequences of legitimacy. For instance, none of the studies in this review considered the links between legitimacy or illegitimacy and the mental health and academic issues confronting children and youth. One study found that being stopped by the police was linked to diminished educational expectations among youth (Jackson et al., 2022) and that procedural justice can buffer the hostile experience of police encounters on the post-traumatic stress symptoms of youth (Gearhart et al., 2023). These two recently published papers were excluded from this review because they were not within the publication period initially set in the search criteria. Finally, consistent with the view of Sun and Wu (2022) that disadvantageous populations other than ethnic minority groups should be included in future research, we argue that future studies could further examine the perceptions of police legitimacy of minority groups (e.g., LGBTQ+, individuals with mental and learning disabilities, and new immigrants) and police self-legitimacy (i.e., officers’ belief about their own legitimacy).

## 5 Limitations

Several limitations of this scoping review warrant acknowledgment. Firstly, this review synthesizes, categorizes and discusses findings from previous studies based on various conceptualizations of police legitimacy, rather than relying on a single model of knowledge. Caution should be exercised when generalizing the results and considering their practical implications. Secondly, it is susceptible to selection bias, primarily because it exclusively incorporated publications in English, thereby potentially overlooking research emanating from non-English-speaking regions, such as Africa and Asia. Thirdly, the review’s focus solely on empirical studies resulted in the exclusion of other valuable forms of research, such as reflective and theoretical papers. This approach may have inadvertently omitted seminal contributions, including the theoretical discourse presented by Tyler and Blader (2003). Fourthly, the review’s reliance on major databases for literature retrieval precluded the inclusion of non-journal publications. Notably, works like the working paper by Murphy and Gaylor (2010), which explores procedural justice’s impact on youth cooperation with police, may have been overlooked. Fifthly, studies featuring age groups outside the predefined scope of this review (i.e., ages 7–18) were excluded, such as research involving young individuals up to the age of 30 in London, as demonstrated by Bradford (2014). Additionally, limitations in the keyword search strategy led to the exclusion of pertinent papers with the title of “injustice,” “illegitimacy,” and “police contact,” for example, a paper discussing procedural injustice by Reisig et al. (2018), and the influence of police contact on urban adolescents’ educational attainment, as evidenced by Gottlieb and Wilson (2019). Sixthly, studies examining procedural justice assessments not exclusively focused on police officers, such as longitudinal research evaluating serious adolescent offenders’ perceptions of procedural justice within both police and court contexts, were not integrated into the review (Kaiser and Reisig, 2019). Finally, the review may have missed recent publications due to the inherent time lag in updating databases like Scopus, as noted by Quvae (2024). For instance, recent works like the study on the impact of police videos on youth’s willingness to cooperate with law enforcement by Tom et al. (2024) may not have been included.

## 6 Conclusion

This scoping review is intended to offer readers a comprehensive overview of the understanding of police legitimacy and procedural justice among individuals aged between 7 and 18. It addresses four research questions (1) How can we *define* police legitimacy and procedural justice for children and youth? (2) What are the *determinants* of police procedural justice and legitimacy for children and youth? (3) What are the *consequences* of police procedural (in)justice and (il)legitimacy for children and youth? (4) Among children and youth, who are the *vulnerable groups* receiving less legitimate and unjust treatment from the police? The findings provide a basis for further discussion of the definition and conceptualization of police legitimacy and have implications for designing feasible interventions that consider the determinants and consequences of police legitimacy. This review also provides a foundation for future research.

## Data Availability

The original contributions presented in the study are included in the article/supplementary material, further inquiries can be directed to the corresponding author.
